# Spontaneous cellular senescence and stress response heterogeneity in human vestibular schwannomas

**DOI:** 10.1093/noajnl/vdag190

**Published:** 2026-07-22

**Authors:** Sandra Franco-Caspueñas, Carmen Ruiz-García, Julio Contreras, Asier Iturrate, Lourdes Rodríguez-De la Rosa, Luis Lassaletta, Isabel Varela-Nieto, Ana M Jiménez-Lara

**Affiliations:** Neurobiology of Hearing and Myelinopathies Group, Institute for Biomedical Research Sols-Morreale (IIBM), Spanish National Research Council (CSIC), Autonomous University of Madrid (UAM), Madrid, Spain; Rare Disease Network Biomedical Research Centre (CIBERER), Institute of Health Carlos III (ISCIII), Madrid, Spain; Hospital La Paz Institute for Health Research (IdiPAZ), Madrid, Spain; Neurobiology of Hearing and Myelinopathies Group, Institute for Biomedical Research Sols-Morreale (IIBM), Spanish National Research Council (CSIC), Autonomous University of Madrid (UAM), Madrid, Spain; Department of Otorhinolaryngology, La Paz University Hospital, Madrid, Spain; Hospital La Paz Institute for Health Research (IdiPAZ), Madrid, Spain; PhD Program in Medicine and Surgery, UAM, Madrid, Spain; Neurobiology of Hearing and Myelinopathies Group, Institute for Biomedical Research Sols-Morreale (IIBM), Spanish National Research Council (CSIC), Autonomous University of Madrid (UAM), Madrid, Spain; Rare Disease Network Biomedical Research Centre (CIBERER), Institute of Health Carlos III (ISCIII), Madrid, Spain; Department of Anatomy, School of Veterinary Medicine, Complutense University of Madrid (UCM), Madrid, Spain; Rare Disease Network Biomedical Research Centre (CIBERER), Institute of Health Carlos III (ISCIII), Madrid, Spain; Genetics and Pathophysiological Mechanisms of Congenital Anomalies Group. Institute for Biomedical Research Sols-Morreale (IIBM), Spanish National Research Council (CSIC), Autonomous University of Madrid (UAM), Madrid, Spain; Neurobiology of Hearing and Myelinopathies Group, Institute for Biomedical Research Sols-Morreale (IIBM), Spanish National Research Council (CSIC), Autonomous University of Madrid (UAM), Madrid, Spain; Rare Disease Network Biomedical Research Centre (CIBERER), Institute of Health Carlos III (ISCIII), Madrid, Spain; Hospital La Paz Institute for Health Research (IdiPAZ), Madrid, Spain; Rare Disease Network Biomedical Research Centre (CIBERER), Institute of Health Carlos III (ISCIII), Madrid, Spain; Department of Otorhinolaryngology, La Paz University Hospital, Madrid, Spain; Hospital La Paz Institute for Health Research (IdiPAZ), Madrid, Spain; Neurobiology of Hearing and Myelinopathies Group, Institute for Biomedical Research Sols-Morreale (IIBM), Spanish National Research Council (CSIC), Autonomous University of Madrid (UAM), Madrid, Spain; Rare Disease Network Biomedical Research Centre (CIBERER), Institute of Health Carlos III (ISCIII), Madrid, Spain; Hospital La Paz Institute for Health Research (IdiPAZ), Madrid, Spain; Neurobiology of Hearing and Myelinopathies Group, Institute for Biomedical Research Sols-Morreale (IIBM), Spanish National Research Council (CSIC), Autonomous University of Madrid (UAM), Madrid, Spain; Hospital La Paz Institute for Health Research (IdiPAZ), Madrid, Spain

**Keywords:** bleomycin sensitivity, navitoclax response, p16 upregulation, SASP markers, transcriptomic profiling

## Abstract

**Background:**

Vestibular schwannomas (VS) are rare tumors of the eighth cranial nerve. Their complex etiology and not-well defined molecular basis prompted an investigation into cellular senescence as a contributing factor to tumor biology.

**Methods:**

Human VS and healthy peripheral nerve (PN) tissue samples, along with primary VS cultures, were analyzed using RNA sequencing (RNA-seq), senescence-associated β-galactosidase (SA-β-GAL) activity assays, RT-qPCR, western blotting, immunofluorescence, and cell viability assays.

**Results:**

RNA-seq revealed differential expression of senescence-related genes in VS compared to normal Schwann cells. SA-β-GAL–positive cells were observed in VS tissues but absent in PN. Primary VS cultures exhibited canonical features of senescence, including elevated SA-β-GAL activity, increased p16 protein levels, and upregulation of senescence-associated secretory phenotype (SASP) markers such as *IL8, IL1B, CCL2,* and *MMP3*. The extent of spontaneous senescence varied among VS cultures and correlated with differential sensitivity to bleomycin-induced senescence: cultures with lower baseline SA-β-GAL activity were more responsive to bleomycin. In contrast, cultures with higher spontaneous senescence showed attenuated activation of the p53/p21 pathway and reduced sensitivity. Furthermore, VS cultures with low baseline senescence were susceptible to combined treatment with bleomycin and the senolytic agent navitoclax, which triggered apoptosis via the intrinsic pathway.

**Conclusion:**

Spontaneous cellular senescence in VS is characterized by p16 upregulation and SASP amplification and is associated with impaired p53/p21 signaling and reduced responsiveness to senescence-inducing agents. These findings suggest that the senescent state of VS cells influences their stress response and may inform future strategies aimed at modulating senescence in tumor contexts.

Key Points

- VS exhibit spontaneous senescence linked to p16 upregulation.
- Attenuated p53/p21 signaling underlies VS spontaneous senescence revealed by bleomycin treatment.
- Senescence heterogeneity influences response to senogenic–senolytic combinations.

Importance of the Study

This study reveals that vestibular schwannomas (VS) exhibit a spontaneous cellular senescence phenotype, marked by increased SA-β-galactosidase activity, p16 upregulation, and elevated expression of SASP components—hallmarks of senescence. Importantly, this senescent state is associated with impaired activation of the p53/p21 axis, resulting in reduced sensitivity to therapy-induced senescence. VS cultures with lower baseline senescence were more responsive to combined treatment with senogenic and senolytic agents, suggesting that senescence heterogeneity may influence p53-regulated response to stress. These findings highlight cellular senescence as a biologically relevant feature of VS and suggest that profiling senescence markers could contribute to tumor stratification. Understanding the molecular diversity of senescence in VS may inform the development of targeted therapeutic strategies and improve experimental modeling of tumor behavior. This work provides a foundation for future studies exploring senescence as a modulator of tumor progression and treatment response in benign nerve sheath tumors.

##  

Vestibular schwannomas (VS) are benign tumors of the Schwann cells (SC) of the vestibulocochlear nerve. They occur either sporadically or bilaterally in the context of *NF2*-related schwannomatosis (NF2-SWN), a hereditary tumor predisposition syndrome.^1^ Although traditionally considered rare, recent epidemiological data suggest that the lifetime prevalence of sporadic VS may exceed 1 in 500 individuals, particularly in older populations.^2-5^ Despite their nonmalignant nature, VS can cause significant neurological and otological morbidity due to their proximity to the brainstem.^6^^,^^7^ Apart from “wait and scan” management of patients, current treatment options include microsurgical resection and stereotactic radiosurgery, both associated with risks such as hearing loss and facial nerve dysfunction.^8^^,^^9^ Stereotactic radiosurgery, which induces DNA damage, has become increasingly used, yet fails to halt tumor progression in a subset of cases. In addition, surgical intervention may result in complications such as cerebrospinal fluid leakage, whereas radiosurgery can be associated with transient tumor enlargement.^10^^,^^11^ Importantly, no pharmacological therapies are currently approved for VS.^12^

Cellular senescence is a tightly regulated, cell-intrinsic program that halts the proliferation of damaged or stressed cells, serving as a critical barrier against malignant transformation.^13^^,^^14^ Beyond its role in tumor suppression, senescence also contributes to physiological processes such as tissue remodeling during development. In the inner ear, developmental senescence has been implicated in morphogenesis, and disruptions to this program have been associated with aberrant cochlear formation.^15-20^

Senescent cells are characterized by a permanent cell cycle arrest and undergo profound morphological and metabolic changes, including chromatin reorganization, increased lysosomal activity, and resistance to apoptosis. A hallmark of senescence is the acquisition of a distinct secretory profile known as the senescence-associated secretory phenotype (SASP), which comprises a complex array of pro-inflammatory cytokines, chemokines, and matrix-remodeling enzymes.^21^^,^^22^

While senescence initially acts as a tumor-suppressive mechanism, the long-term persistence of senescent cells can have deleterious effects. Chronic SASP signaling may promote tumor progression, immune evasion, and remodeling of the tumor microenvironment, facilitating processes such as epithelial–mesenchymal transition and stemness acquisition.^23^^,^^24^ These dual roles underscore the importance of understanding senescence dynamics in both physiological and pathological contexts, including tumor biology.

Previous studies have demonstrated that schwannomas arising from various peripheral nerves (PNs) accumulate senescent cells, distinguished by elevated levels of p16 and SA-β-galactosidase (SA-β-GAL) activity, along with reduced expression of proliferation markers such as Ki67. These features clearly differentiate them from malignant PN sheath tumors,^25^ suggesting that cellular senescence may serve as a protective mechanism against malignant transformation in these benign neoplasms. In line with this, HEI-193 human VS cells have been shown to respond effectively to a sequential treatment strategy combining senescence-inducing (senogenic) agents with senolytic compounds, such as navitoclax,^26^^,^^27^ which selectively eliminate senescent cells.^28^ This “one-two punch” approach highlights the potential of targeting senescence as a therapeutic avenue in VS.

Despite these promising observations, the molecular mechanisms governing senescence in VS remain poorly characterized. The extent to which spontaneous senescence contributes to tumor behavior, treatment response, and microenvironmental interactions is still unclear. A deeper understanding of these processes could provide valuable insights into VS pathophysiology and support the development of stratified, biology-driven therapeutic strategies.

Here, we present the study of spontaneous cellular senescence in VS, using both tumor tissue and primary cell cultures. We identified senescent cells in intact VS tumors, confirmed by SA-β-GAL staining, and observed increased expression of senescence-related genes. *In vitro*, primary VS cultures exhibited hallmark features of senescence, including elevated SA-β-GAL activity, p16 upregulation, and induction of SASP components such as *IL8, IL1B, CCL2,* and *MMP3*. The degree of spontaneous senescence varied among cultures and was associated with differential sensitivity to genotoxic agents such as bleomycin. Cultures with lower baseline senescence were more responsive to therapy-induced senescence and to senolytic agents like navitoclax, which selectively eliminated senescent cells via intrinsic apoptotic pathways. In contrast, cultures with high spontaneous senescence showed attenuated activation of the p53/p21 axis and reduced sensitivity to genotoxic stress. These findings suggest that senescence heterogeneity may influence the cellular response to stress and therapeutic agents.

Given the emerging role of senescence in tumor biology, profiling senescence markers in VS could provide a basis for tumor stratification and personalized therapeutic approaches. Senolytic strategies, including PROTACs and galacto-conjugated compounds, have shown promise in enhancing specificity and reducing toxicity.^29^^,^^30^ Our findings support the hypothesis that cellular senescence is a biologically relevant and potentially actionable feature of VS, with implications for both experimental modeling and clinical management.

## Methods

### Human Tissue Collection and Processing

Fresh VS tumor specimens were obtained from patients diagnosed with either sporadic VS or NF2-SWN who underwent surgical resection between 2017 and 2024. Control samples of healthy PN tissue were also collected. The greater auricular nerve (GAN) was used as a source of PN tissue and to establish normal human SC cultures. The nerve was previously sacrificed as part of a facial reanimation procedure (hypoglossal-to-facial nerve anastomosis). All specimens were immediately placed in HBSS supplemented with Ca^2+^/Mg^2+^ (HBSS^+^/^+^; Gibco, Thermo Fisher Scientific, Waltham, MA, USA) and kept on ice to preserve tissue integrity. Following surgical criteria, VS samples—no larger than 5 mm³—were systematically excised from the central region of the tumor to ensure comparability across specimens. When feasible, portions of the same tumor were divided and processed for multiple downstream applications: preserved in RNAlater (Thermo Fisher Scientific) and stored at −80 °C for molecular analyses or fixed and sectioned for histological and immunohistochemical studies.

### Clinical Data Collection and Patient Characterization

Clinical records, surgical reports, preoperative magnetic resonance imaging (MRI), and pathology reports were reviewed for all patients included in this study. A translabyrinthine approach was performed in all cases.

Patient data were annotated using standardized criteria, including age at diagnosis, sex, clinical presentation, preoperative hearing status, tumor dimensions and volume, and *NF2* mutation status in blood and/or tumor tissue. A summary of these variables is provided in Table 1. Hearing loss was classified into 4 grades (A-D) according to the guidelines of the American Academy of Otolaryngology–Head and Neck Surgery.^31^ Tumor volume was calculated from MRI scans following previously established protocols.^31^^,^^32^ Representative MRI images from six patients are shown in Supplementary Figure S1.

**Table 1.** Patient demographics, clinical and genetic characteristics

ID	Age at diagnosis (years)	Gender	Clinical presentation	Preoperative hearing^a^	Tumoral size (diameter, mm)^b^	Tumoral size (cc)^b^	*NF2* status (blood)	*NF2* status (tissue)^c^
VS2^#^	61	Female	Facial paralysis, dizziness	B	27	5.82	Negative	c.849delC PV*
VS4^#^	52	Female	Hearing loss, dizziness	C	18	1.50	Negative	c.566_578delCTGCTTGGTACGC PV*
^d^VS17^#^	23	Female	Hearing loss	A	40	20.64	c.1446 + 1G>A *PV*	c.1446 + 1G>A *PV*
VS20^#^	45	Male	Hearing loss, dizziness, tinnitus	A	30	7.94	Negative	c.658delA PV*
VS21^#^	56	Female	N/A	N/A	22	3.96	ND	Negative
VS22^#^	43	Female	Hearing loss, tinnitus, aural fullness	B	25.8	6.91	Negative	Negative
VS23	49	Female	Hearing loss, tinnitus	C	26	7.82	ND	ND
VS24^#^	52	Male	Hearing loss, tinnitus, dizziness	A	34.5	13.22	ND	Negative
VS25^#^	73	Male	Imbalance, traumatic brain injury	D	48	24.17	N/A	Negative
VS26	38	Female	Facial numbness, headache	D	26	7.78	Negative	N/A
VS27^#^	35	Male	Hearing loss, headache	C	36.4	22.41	ND	c.321delG; c.1002delG PV*; PV*
VS30^#^	69	Male	Hearing loss, imbalance	D	35	14.17	Negative	c.1069_1070delGA* PV*
VS31^#^	44	Female	Hearing loss, dizziness, headache, facial paralysis	C	34.4	10.76	Negative	c.432C>G *PV*
VS50	37	Male	Tinnitus, hearing loss	C	28	6.27	Negative	ND
VS55	53	Female	Tinnitus, hearing loss, dizziness	B	37	20.88	Negative	ND
VS60	72	Female	Worsening hearing loss, dizziness	D	30	12.75	Negative	ND
VS62	48	Male	Sudden hearing loss, tinnitus	A	31.5	13.24	Negative	ND
VS63	49	Male	Hearing loss	B	35	15.94	Negative	c.1613_1638del PV*
VS72	18	Female	Hearing loss	D	N/A	N/A	Negative	c.193C>T; c.600-8_600-2delCCCCACA PV; PV*
VS73	63	Male	Imbalance, hearing loss	C	35	17.08	Negative	c.715delC: p.Leu239Trpfs*12 PV*
VS76	57	Female	Dizziness, tinnitus, mild hearing loss	A	19	2.36	ND	p.Tyr153Ter LPV*
^d^VS78	56	Female	Hearing loss and imbalance. Previous right VS	C	12	0.3	Negative	c.448-1G>A; c.1219C>T *PV; PV*
VS79	57	Female	Hearing loss and tinnitus	D	32	12.19	Negative	c.240 + 2T>G PV*
VS80	29	Female	Hearing loss, tinnitus and headache	D	25	3.89	Negative	c.363G>T; c.743delA LPV*; PV*

Clinical information of patients diagnosed with vestibular schwannoma (VS) includes age at diagnosis, gender, clinical presentation, preoperative hearing, tumor measurements and volume, and *NF2* status in blood and tissue.

a Preoperative hearing was classified according to the American Academy of Otolaryngology–Head and Neck Surgery into four grades based on pure tone average (PTA) and the speech discrimination score (SDS): A, PTA < 30 dB, SDS 70%-100%; B, PTA < 31-50 dB, SDS 50%-69%; C, PTA > 50 dB, SDS 50%-69%; D, any PTA, SDS < 50%.

b Tumor volume was calculated on Magnetic Resonance Imaging (MRI).

c Variant classification was performed according to ACMG recommendations. Variant marked with an asterisk (*) does not appear in ClinVar but is classified as pathogenic/likely pathogenic according to its relationship to clinical manifestations.

d Patients diagnosed with *NF2*-related schwannomatosis. The remaining patients have a sporadic VS. The number symbol (#) indicates tumor samples were used in the RNA-seq experiment described in this study. PV: Pathogenic variant; LPV: Likely pathogenic variant; ND: not determined; N/A: not available.

Genetic analyses to detect *NF2* gene alterations were performed by the Genetics Unit at Hospital La Paz, using blood and/or tumor tissue samples. One patient (VS30) had previously received radiotherapy.

### RNA-Seq Gene Expression and Bioinformatic Analysis

VS samples preserved in RNAlater at −80 °C were processed for RNA extraction using QIAzol lysis reagent and the RNeasy Mini Kit (QIAGEN GmbH, Hilden, Germany), following the manufacturer’s protocol. RNA from human Schwann cells (SC; ScienCell, Carlsbad, CA, USA) was used as a reference control. Only RNA samples with an integrity number (RIN) greater than 7.0 were selected for sequencing.

RNA-seq analysis was performed on 11 VS samples (VS2, VS4, VS17, VS20, VS21, VS22, VS24, VS25, VS27, VS30, and VS31; Table 1) and SC RNA controls at the Madrid Science Park using the Illumina HiSeq 4000 platform (Illumina, San Diego, CA, USA). Sequencing reads were first aligned to the human reference genome (hg38, GRCh38) using TopHat. Gene expression levels were quantified and normalized as fragments per kilobase of transcript per million mapped reads (FPKM). Quality control filtering was applied according to platform-specific guidelines.

Differentially expressed genes (DEGs) between VS and reference SC samples were identified based on fold change (FC > 2 or < 0.5) and statistical significance (q-value < 0.05). Functional enrichment analysis of DEGs was conducted using the Metascape bioinformatics platform, including Gene Ontology (GO) term categorization. Gene Set Enrichment Analysis was performed to identify enriched gene sets. The false discovery rate (FDR) was used to assess statistical significance, and only gene sets with FDR < 0.05 were considered significant and included in downstream analyses. Pathway enrichment was specifically filtered for the KEGG senescence pathway (hsa04218). Protein–protein interaction (PPI) networks among these genes were analyzed using the STRING database and visualized using Cytoscape version 3.9.1.

Microarray gene expression data from Torres-Martin, et al.^33^ were retrieved from NCBI GEO database (GSE39645). Differential expression analysis between VS and SC was conducted using NCBI GEO2R tool (https://www.ncbi.nlm.nih.gov/geo/geo2r/). Probe-to-gene mapping was performed using a custom R pipeline, and for those genes mapping to several probes, the probe with the highest absolute log2 fold change (log2FC) was retained. DEGs were defined by an adjusted *P*-value < .05 and |log2FC| > 1.

### Cell Culture and Treatments

Primary human VS cell cultures were established as previously described.^34^ Cells were maintained in Dulbecco’s Modified Eagle’s Medium (DMEM; Gibco) supplemented with 10% fetal bovine serum (FBS; Biowest, Nouaillé, France, EU), 0.05% gentamicin (Normon Laboratories, Madrid, Spain), 10 ng/mL Neuregulin-1 (Nrg-1; R&D Systems, Minneapolis, MN, USA), and 0.2 µM forskolin (Cayman Chemical, Ann Arbor, MI, USA). Cultures were incubated at 37 °C in a humidified atmosphere with 5% CO_2_ on plates pre-coated with 0.01% poly-L-ornithine and 10 µg/mL laminin (Sigma-Aldrich, Saint Louis, MO, USA). All experiments using primary VS cells were performed at passage 1.

Human SC were also isolated by following the same protocol with specific modifications. Briefly, PN samples (approximately 2 cm in length) were dissected under a microscope, and nerve fascicles were cut into 1-2 mm segments. These were incubated for 15 days in complete SC medium (DMEM supplemented with 10 ng/mL Nrg-1 and 2 µM forskolin) to promote SC dedifferentiation prior to enzymatic digestion, as previously described.^34^^,^^35^ The purity of primary cultures was confirmed by immunolabeling for S100B, a marker of the SC lineage.

The immortalized HEI-193 cell line, derived from a VS tumor of a patient with NF2-SWN, was used as a reference model. Cells were cultured in DMEM GlutaMAX™ (Gibco) supplemented with 10% FBS and 1% (v/v) penicillin/streptomycin (Gibco), as previously described.^28^^,^^36^

Cells were treated with bleomycin (Mylan, Viatris, Canonsburg, PA, USA), dissolved in 0.9% NaCl, or with navitoclax (NAVI, ABT-263; Selleckchem, Houston, TX, USA), dissolved in DMSO. Bleomycin is a glycopeptide antibiotic that induces DNA single- and double-strand breaks at the 3′-4′ bonds of deoxyribose. Navitoclax is a small-molecule inhibitor that selectively binds to anti-apoptotic proteins BCL-2, BCL-XL, and BCL-w, which are frequently overexpressed in various cancers.^26^ The final concentration of DMSO in the culture medium did not exceed 0.1% of the total volume.

### Measurement of Cellular Viability and Detection of Senescence-Associated *β*-Galactosidase Activity

Primary cells (VS23, VS25, VS26, VS50, VS55, VS60, VS62, VS63, VS73, VS76, and VS79) were seeded in 48-well flat-bottom plates and incubated for 48 hours. They were then fixed with 1% (w/v) glutaraldehyde (Merck, Kenilworth, NJ, USA) for 10 minutes, washed with 0.01 M phosphate-buffered saline, pH 7.4 (PBS; Sigma-Aldrich), and stained with 0.1% (w/v) crystal violet solution (Merck) for 30 minutes. After extensive rinsing with deionized water, cells were air-dried, and the dye was solubilized in 10% (v/v) acetic acid. Optical density was measured at 590 nm using a VERSAmax™ tunable microplate reader with SOFTmax® Pro 3.0 software (Molecular Devices, San José, CA, USA). Mean absorbance from vehicle-treated control wells was considered 100% cell viability. Additionally, cell viability was assessed by trypan blue exclusion (Gibco).

VS tumor and PN tissue samples were washed once with PBS and fixed with 2% formaldehyde/0.2% glutaraldehyde (Merck). After washing, samples were incubated for 18 hours at 37 °C in a staining solution containing 20 mg/mL X-Gal (prepared in dimethylformamide), 0.2 M citric acid/Na phosphate buffer (pH 6), 100 mM potassium ferrocyanide, 100 mM potassium ferricyanide, 5 M sodium chloride, and 1 M magnesium chloride (all from Sigma-Aldrich), with gentle shaking and protected from light.^37^ After incubation, samples were washed in PBS, dehydrated in isopropanol to preserve SA-β-GAL staining, and embedded in paraffin. Serial 5 µm sections were cut using a Leica RM2155 microtome, rehydrated in isopropanol and graded ethanol solutions, and counterstained with 0.5% nuclear red in distilled water (Panreac, ITW Reagents, Monza, Italy). Brightfield images were acquired using a DP70 camera (Olympus) mounted on an Axiophot microscope (Zeiss). Quantification of blue-stained areas, indicative of senescent cells, was performed using ImageJ v1.52a software (NIH, Bethesda, MD, USA). Twenty fields per tumor were analyzed, and results were expressed as a percentage of the total tissue area, which was considered 100%.

For cultured cells, primary VS cultures (VS23, VS25, VS26, VS50, VS55, VS60, VS62, VS63, VS72, VS73, VS76, VS78, VS79, and VS80) were fixed with 2% formaldehyde/0.02% glutaraldehyde in PBS for 4 minutes, washed, and incubated with the same X-Gal staining solution for 18 hours at 37 °C, protected from light. After staining, cells were washed with PBS and visualized using a LEICA DMi1 microscope equipped with a FLEXACAM C1 camera (Leica Microsystems, Wetzlar, Germany).

### Western Blotting and Gene Expression Analysis by RT-qPCR

Antibodies against caspase-3, caspase-8, p21, γH2AX, and phospho-p53 (all from Cell Signaling Technology), β-actin-HRP (BACT; Sigma-Aldrich), p16 (Abcam, Cambridge, United Kingdom), and p53 and vinculin (VCL) (both from Santa Cruz Biotechnology, Santa Cruz, CA, USA) were used for western blotting analysis (Supplementary Table S1) as previously described.^28^ Immune complexes were detected by chemiluminescence using the Clarity™ Western ECL Substrate (Bio-Rad, Hercules, CA, USA) and visualized on a Fusion-Solo.6S EDGE V0.70 chemiluminescence imaging system (Vilber, Marne-la-Vallée, France). Protein levels were normalized to BACT or VCL loading controls and quantified using ImageJ software (NIH, Bethesda, MD, USA).

Total RNA was extracted using the SpeedTools Total RNA Extraction Kit (BioTools, Madrid, Spain). For reverse transcription, 0.5 µg of RNA was used with the iScript cDNA Synthesis Kit (Bio-Rad). Quantitative PCR (RT-qPCR) was performed using FS Universal SYBR Green Master Mix (Applied Biosystems/Thermo Fisher Scientific, Waltham, MA, USA) and gene-specific primers (sequences available upon request) on a CFX96 Touch Real-Time PCR thermal cycler (Bio-Rad). For Supplementary Figure 3A, gene expression was analyzed using Taqman probe-based assays at the IIBM Genomics Core Facility. Expression levels were determined in 10 VS samples (VS2, VS4, VS17, VS20, VS21, VS22, VS24, VS25, VS27, and VS31; Table 1) and compared with control SC RNA. Relative gene expression changes were calculated using the 2(^−ΔΔCT^) method and normalized to the housekeeping gene *18S* ribosomal RNA or *HPRT1*, as specified in figure legends.

### Immunofluorescence Studies

Primary VS (VS55 and VS62), HEI-193 or SC cells were seeded onto 12-mm diameter round glass coverslips placed in a 24-well plate and cultured for 2 days. Cells were then fixed for 15 minutes at RT in 3.7% formaldehyde (Sigma-Aldrich) and washed twice with PBS. Permeabilization was performed for 20 minutes using 0.5% Triton X-100 (Sigma-Aldrich). After washing, the slides were blocked for 30 minutes in PBS containing 0.1% Triton X-100 (Sigma-Aldrich), 5% normal goat serum (NGS; Sigma-Aldrich), and 3% BSA (NZYTech). Primary antibodies were diluted in PBS containing 0.1% Triton X-100, 1% NGS, and 3% BSA and incubated overnight at 4 °C. Slides were then washed several times with PBS containing 0.075% Tween 20 (Sigma-Aldrich), followed by incubation with a secondary antibody (Alexa Fluor®-conjugated goat anti-mouse IgG; Thermo Fisher Scientific) at a 1:300 dilution for 2 hours at RT. Finally, slides were washed three times with PBS-0.075% Tween 20, stained with DAPI, and mounted using ProLong® Diamond Antifade Reagent (Thermo Fisher Scientific). Primary antibodies used are listed in Supplementary Table S1. Fluorescence images were acquired using an inverted Zeiss LSM710 laser scanning confocal microscope (Zeiss) equipped with a 40x/1.3 Plan-Apochromatic oil immersion objective. Each channel was acquired sequentially. Confocal images shown are two-dimensional maximum intensity projections of z-series stacks. Images were acquired using Zen 2.3 SP1 software (Zeiss) and processed with ImageJ (NIH, Bethesda, MD, USA).

### Statistical Analysis

Statistical analyses were performed using GraphPad Prism 10 software (GraphPad Software, San Diego, CA, USA) and R (version 4.5.2; R Foundation for Statistical Computing, Vienna, Austria) within the RStudio IDE (Posit Software, PBC, Boston, MA, USA). Data are presented as mean ± SD from three independent cultures, as indicated in figure legends. For HEI-193 cells, data represent the mean ± SD from at least three independent experiments, each performed in triplicate. Normality and homoscedasticity were assessed using the Shapiro–Wilk and Levene tests, respectively. Comparisons between 2 groups were performed using Student’s *t*-test for normally distributed data or the Mann-Whitney test for non-parametric data. For multiple group comparisons, one- or two-way ANOVA was applied when parametric assumptions were met, followed by the appropriate post hoc tests. When normality was not met, non-parametric alternatives were used (Kruskal–Wallis or aligned rank transform ANOVA). Correlations were analyzed using Pearson’s correlation coefficient. Statistical significance was set at *P *< .05 (*P *< .05, *P < .01, **P < .001).

## Results

### VS Tumors Exhibit Features of Cellular Senescence

**Figure 1.**
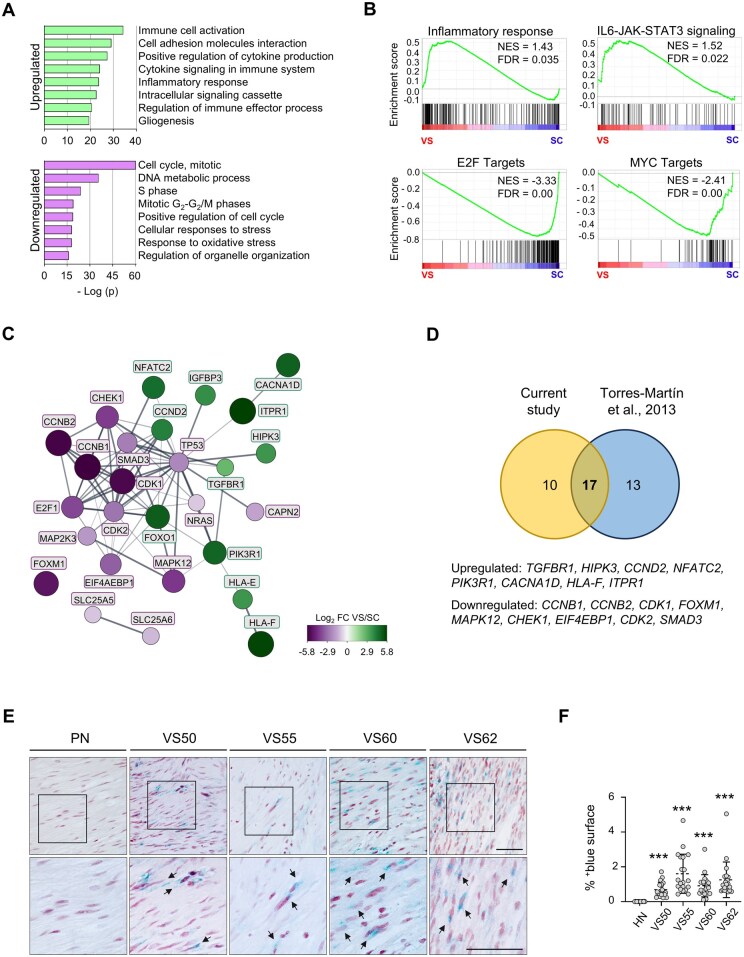
Cellular senescence in human vestibular schwannoma (VS). (A) Functional enrichment analysis of differentially expressed genes (DEGs) in VS against Schwann cells (SC) by using Metascape revealed upregulated and downregulated biological pathways. (B) Gene Set Enrichment Analysis (GSEA) performed on the transcriptomic profiles of VS and SC cells, showing relevant biological signatures and signaling pathways that are differentially enriched between the two conditions. (C) Network for protein–protein interaction of DEGs in VS using a cellular senescence KEGG pathway (hsa04218). (D) Venn diagram showing the overlap of DEGs identified after intersecting each dataset with the KEGG cellular senescence pathway (hsa04218) in the study by Torres-Martín et al. and in the current study, both of which used SC RNA as a control. Representative examples of the 17 overlapping genes, including both upregulated and downregulated genes, are shown. (E) Representative brightfield optical microscopy images of a human peripheral nerve (PN) and VS tissue samples sections stained to analyze SA-β-GAL activity (perinuclear blue staining) and counterstained with neutral red. Black arrows on right panels indicate spindle SA-β-GAL positive cells (scale bar = 50 µm). (F) The graph shows the quantification of blue area in each sample section. Data corresponds to the mean ± SD of 20 different fields. Statistical significance was determined by Kruskal-Wallis test. Symbols denote statistically significant differences: *P < .05; **P < .01; ***P < .001.

The initial objective was to identify signaling pathways and genes related to cellular senescence that are differentially expressed in VS compared to normal SC. To this end, we analyzed RNA-seq data from a representative cohort of VS samples (Table 1). A total of 1,975 genes were found to be significantly dysregulated in VS. To explore senescence-related gene expression, we applied multiple analytical approaches. Pathway enrichment analysis revealed that cytokine signaling and inflammatory response pathways were among the most enriched in the VS gene set, while genes involved in cell cycle regulation were notably downregulated (Figure 1A). In addition, we performed GSEA to the RNA-seq dataset to further validate pathway-level changes identified in Metascape analysis. GSEA revealed significant positive enrichment of hallmark gene sets associated with inflammatory signaling, including the inflammatory response and IL6–JAK–STAT3 signaling, indicating activation of these programs. In contrast, gene sets related to proliferation, such as E2F and MYC target genes, showed negative enrichment, consistent with cell cycle inhibition (Figure 1B). These results reinforce independently the transcriptional shift toward enhanced inflammatory signaling and reduced proliferation in VS. We then cross-referenced the DEGs with the KEGG senescence pathway database. This analysis revealed significant upregulation of known senescence inducers, alongside downregulation of several senescence inhibitors and proliferation-related genes (Figure 1C). In total, 27 genes showed significant differential regulation (q-value < 0.05) in VS compared to SC, which were also validated by qRT-PCR (Supplementary Figure S3A), supporting the definition of a functionally coherent subset associated with senescence-related transcriptional changes. The most significantly altered transcripts, categorized by pathway and fold change, are listed in Supplementary Table S2. To further contextualize our RNASeq findings, we assessed the degree of overlap between DEGs associated with the KEGG senescence signaling pathway between the publicly available dataset (GSE39645) and our RNA-Seq data. The results, presented as a Venn diagram in Figure 1D, show that 17 genes are shared between the two studies, while 10 genes are uniquely identified in the current dataset and 13 are specific to GSE39645, in which SC were used as controls. The shared genes identified include key regulators of cell-cycle progression (eg *CCNB1*, *CCNB2*, *CDK1*, *CDK2*, *FOXM1*) and components of signaling pathways (eg *TGFBR1*, *PI3KR1*), suggesting selective engagement of core regulatory modules potentially associated with a senescence program. In addition, re-analysis of GSE39645 using SC as control yielded a similar enrichment pattern (Supplementary Figure S3B-C), further supporting the consistency and reliability of these findings across independent datasets.

To validate these findings at the tissue level, paraffin-embedded sections of VS and PN samples were analyzed for SA-β-GAL activity—a canonical marker of cellular senescence—using histochemical staining. As shown in Figure 1E (upper panels), SA-β-GAL-positive cells were variably present in hypercellular regions of VS tumors, whereas no staining was observed in PN samples. Moreover, SA-β-GAL-positive cells in VS sections displayed the characteristic spindle-shaped morphology of schwannoma cells (Figure 1E, lower panels). Quantification of the stained area across 20 random fields per tumor revealed substantial variability in the number of SA-β-GAL-positive cells (Figure 1F). Taken together, these results suggest that spontaneous cellular senescence may potentially play a role in the pathobiology of VS.

### Primary VS Cell Cultures Show Elevated SA-*β*-GAL Activity and Increased p16 Protein Levels

**Figure 2.**
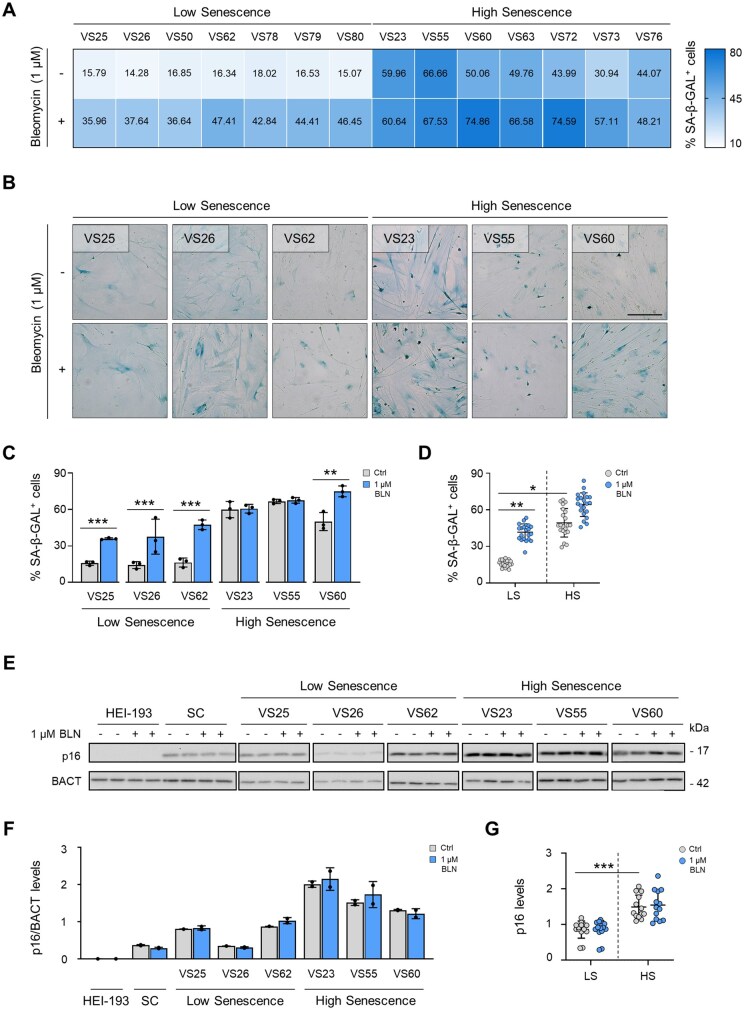
Primary VS cultures show hallmarks of senescence: SA-β-GAL activity can be increased by pro-senescence inducers. (A) Quantification of SA-β-GAL activity of 14 different control and bleomycin-treated (1 µM, 5 days) primary VS cultures. Percentage of SA-β-GAL positive cells is shown in a heatmap, blue-highest, white-lowest, as illustrated at the right side of the figure. Data correspond to the mean of three independent primary VS cultures. (B) Representative brightfield images of control or bleomycin-treated (1 µM, 5 days) primary VS cells (scale bar = 200 µm). (C) Quantification of SA-β-GAL positive control or bleomycin-treated (1 µM, 5 days) primary VS cells shown in B. Data correspond to the mean ± SD of 3 independent primary VS cultures. (D) SA-β-GAL percentages shown in panel A are plotted by clustering the different primary cell lines according to their senescence features: low senescence (LS: VS25, VS26, VS50, VS62, VS78, VS79, and VS80) and high senescence (HS: VS23, VS55, VS60, VS63, VS72, VS73, and VS76). Data correspond to the mean ± SD of 7 different cultures performed by triplicate. (E) Protein levels of p16 were measured by western blotting in control and bleomycin-treated (1 µM, 5 days) primary VS cells, immortalized HEI-193 cells and SC. ß-actin was used as a loading control. (F) Quantification of p16 protein levels found in control and bleomycin-treated (1 µM, 5 days) cells shown in E. Values are normalized to ß-actin. Data correspond to the mean ± SD of 2 independent samples. (G) Data are plotted as described in D and correspond to the mean ± SD of 7 different primary cell lines performed by duplicate. Statistical significance was determined by Student′s t-test (C) and Aligned Rank Transform procedure to perform a non-parametric two-way ANOVA (D, G). Symbols denote statistically significant differences: *P < .05; **P < .01; ***P < .001.

We next investigated whether SA-β-GAL-positive cells could also be detected in primary cultures derived from VS. Fourteen primary VS cultures were analyzed for SA-β-GAL activity. We observed that between 15% and 70% of cells in these cultures were SA-β-GAL-positive (Figure 2A). To assess whether senescence in these cells could be further induced, we treated the cultures with bleomycin, a DNA-damaging agent. Bleomycin treatment led to an overall increase in the proportion of SA-β-GAL-positive cells across the cultures analyzed (Figure 2A). A representative set of 6 primary VS cultures with high (VS23, VS55, and VS60) and low (VS25, VS26, and VS62) basal levels of SA-β-GAL-positive cells is shown in Figure 2, panels B and C. The fourteen VS primary cultures were categorized into two groups based on their baseline levels of SA-β-GAL-positive cells: low and high senescence, LS and HS, respectively. We first confirmed that the percentage of SA-β-GAL-positive cells was significantly different between the two groups. Bleomycin treatment increased SA-β-GAL activity primarily in LS cultures, while HS cultures showed no further induction, suggesting that the senescence status of VS cells may influence their responsiveness to DNA damage (Figure 2D).

We then examined the expression of the tumor suppressor protein p16 in the representative set of primary VS cultures. As shown in Figures 2E-F, p16 protein levels were elevated in all primary VS cultures, and to a lesser extent, in normal SC. In contrast, HEI-193 cells showed no detectable p16 expression. Furthermore, HS cultures exhibited significantly higher p16 levels compared to LS cultures (Figure 2G). As expected, bleomycin treatment did not alter p16 protein levels in any of the cultures analyzed (Figure 2G). In addition, a strong positive linear correlation was observed between basal percentage of SA-β-GAL-positive cells and p16 protein levels (R = 0.7999, *P* < .001), consistent with the hypothesis that p16 may contribute to the acquisition or maintenance of the senescent phenotype (data not shown).

### HS Primary Cultures Exhibit an Inflammatory Gene Expression Profile Associated with Cellular Senescence

We next compared the expression of a panel of pro-inflammatory genes associated with the SASP in the initial set of 6 primary VS cultures relative to normal SC. Immortalized HEI-193 cells were also included in the analysis. Gene expression was evaluated under control conditions and following bleomycin treatment.

**Figure 3.**
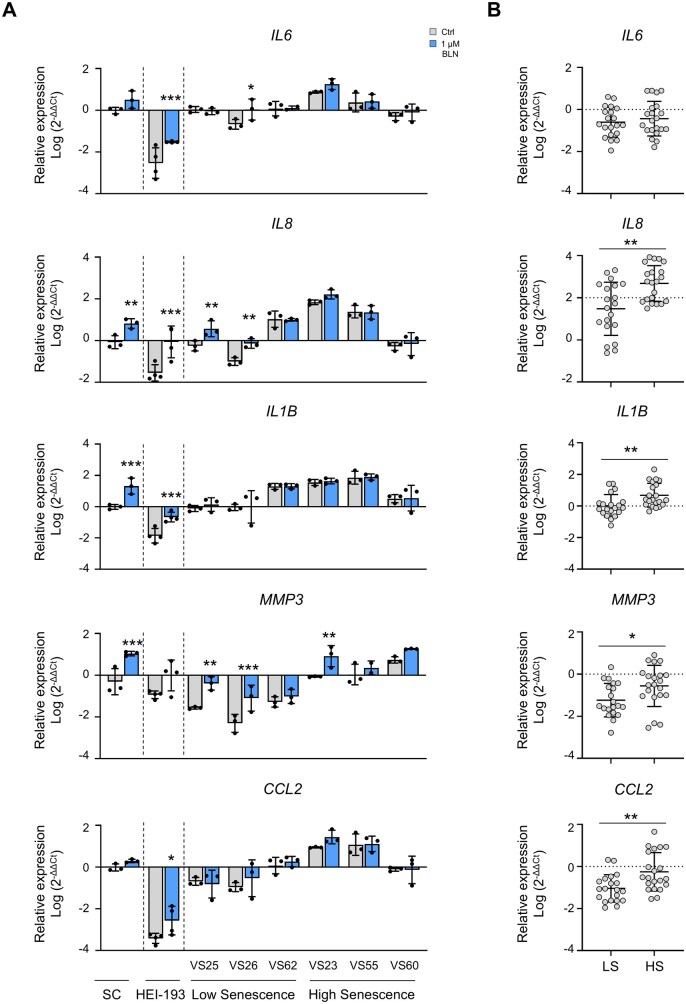
Primary VS cultures with high senescence display a proinflammatory gene expression pattern. (A) RT-qPCR assessment of *IL6, IL8, IL1B, CCL2,* and *MMP3* normalized to *18S* levels in control or bleomycin-treated (1 µM, 5 days) primary VS cells, all related to the expression in control SC. Data correspond to the mean ± SD of 3 independent primary cultures, or four independent experiments made by triplicate in the case of HEI-193 cells. (B) SASP gene expression levels are plotted by clustering the different primary cell lines according to their senescence features: low senescence (LS: VS25, VS26, VS50, VS62, VS78, VS79, and VS80) and high senescence (HS: VS23, VS55, VS60, VS63, VS72, VS73, and VS76). Data correspond to the mean ± SD of 7 different cultures performed by triplicate. Statistical significance was determined by two-way ANOVA (A) and Student’s t-test or Mann-Whitney test (B). Symbols denote statistically significant differences against the values found against their control conditions: *P < .05; **P < .01; ***P < .001.

Expression levels of *IL6, IL8, IL1B, MMP3,* and *CCL2* genes in control conditions varied among primary VS cultures but were consistently higher in HS cultures (VS23, VS55, and VS60) compared to LS cultures (VS25, VS26, and VS62), whose expression profiles were more similar to those of SC. Immortalized HEI-193 cells exhibited significantly lower expression of these genes in control conditions than primary VS cultures (Figure 3A). Upon bleomycin treatment, SC and HEI-193 cells showed a marked upregulation of pro-inflammatory genes. In contrast, only a subset of primary VS cultures responded to bleomycin with significant increases in *IL8* and *MMP3* expression (Figure 3A). When the fourteen primary VS cultures were grouped based on their senescence status, we found that expression in control conditions of *IL8, IL1B, MMP3,* and *CCL2* was significantly higher in HS cultures than in LS cultures (Figure 3B), indicating a stronger pro-inflammatory signature in cultures with a higher proportion of senescent cells.

### Lack of Bleomycin-Induced Senescence in HS Primary Cultures Is Associated with Attenuated p53/p21 Activation

To investigate the molecular basis underlying the differential response to bleomycin in the two subtypes of primary VS cultures, we assessed genomic damage by measuring γH2AX levels and evaluated activation of the p53/p21 pathway following bleomycin treatment or under control conditions.

**Figure 4.**
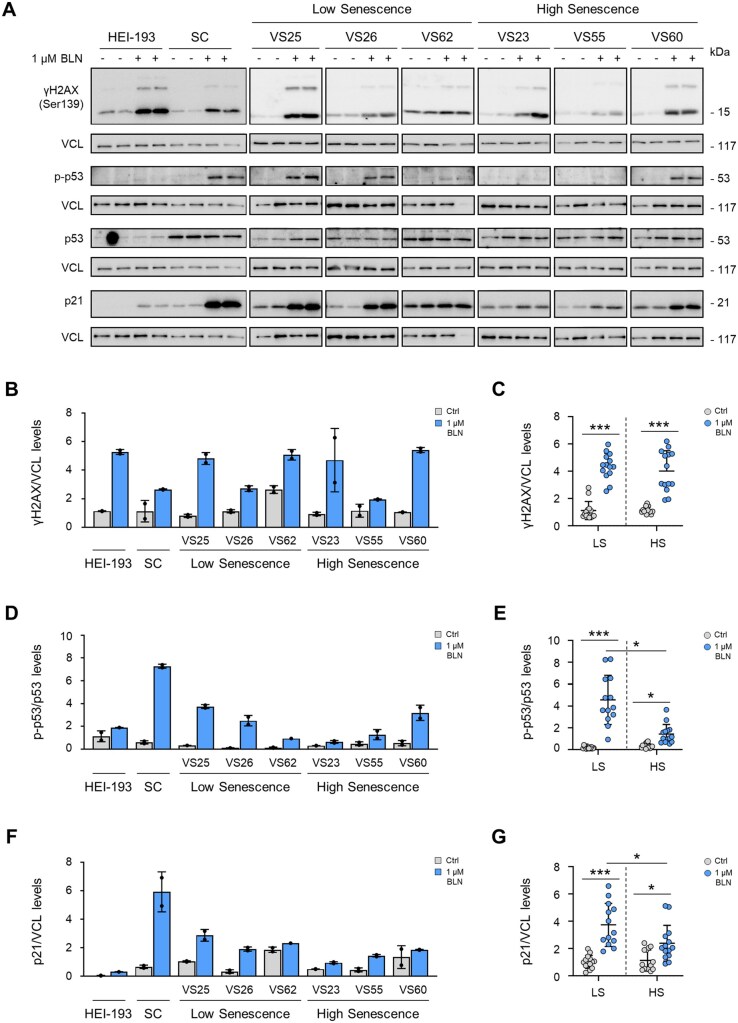
Spontaneous cellular senescence is associated with reduced activation of the p53/p21 axis following a DNA damage stimulus in primary VS cultures. (A) Protein levels of γH2AX, phospho-p53, p53, and p21 were measured by western blotting in control or bleomycin-treated (1 µM, 5 days) primary VS cells, immortalized HEI-193 cells and SC. Vinculin was used as a loading control. (B) Quantification of γH2AX protein levels found in control and bleomycin-treated (1 µM, 5 days) cells shown in A. Values are normalized to vinculin. A shorter exposure time was needed for the immortalized HEI-193 cells and SC than for the primary VS samples. Data correspond to the mean ± SD of 2 independent samples. (C) γH2AX protein levels are plotted by clustering the different primary cell lines according to their senescence features: low senescence (LS: VS25, VS26, VS50, VS62, VS78, VS79, and VS80) and high senescence (HS: VS23, VS55, VS60, VS63, VS72, VS73, and VS76). Data correspond to the mean ± SD of seven different primary cell lines performed by duplicate. (D) Quantification of the phospho-p53/p53 protein ratio found in control and bleomycin-treated (1 µM, 5 days) cells shown in A. Values are normalized to vinculin. Data correspond to the mean ± SD of 2 independent samples. (E) Phospho-p53/p53 protein ratio data are plotted as described in panel C. Data correspond to the mean ± SD of seven different primary cell lines performed by duplicate. (F) Quantification of p21 protein levels found in control and bleomycin-treated (1 µM, 5 days) cells shown in A. Values are normalized to vinculin. Data correspond to the mean ± SD of two independent samples. (G) p21 protein levels are plotted as described in panel C. Data correspond to the mean ± SD of 7 different primary cell lines performed by duplicate. Statistical significance was determined by Aligned Rank Transform procedure to perform a non-parametric factorial two-way ANOVA. Symbols denote statistically significant differences: *P < .05; **P < .01; ***P < .001.

Immunofluorescence (Supplementary Figure S2) and western blot analysis (Figure 4A) revealed detectable γH2AX levels in primary VS cultures, comparable to those observed in immortalized HEI-193 cells and normal SC. Bleomycin treatment further increased γH2AX levels in all cell types, including primary VS cultures, HEI-193 cells, and SC (Figures 4A-B). No significant differences in γH2AX induction were observed between LS and HS cultures, indicating that both subtypes are equally susceptible to DNA damage (Figure 4C).

To determine whether the p53/p21 axis was activated in response to bleomycin, we analyzed phosphorylated p53, total p53, and p21 protein levels—a downstream target of p53—in primary VS cultures, HEI-193 cells, and SC. Bleomycin treatment induced phosphorylation of p53 in SC and LS cultures but was significantly diminished in HS cultures, which showed poor activation of p53 following treatment (Figures 4A, D, E). p21 protein levels in control conditions were higher in primary VS cultures compared to HEI-193 cells and SC (Figure 4A, F), although no differences were observed between LS and HS cultures under control conditions. Upon bleomycin treatment, LS cultures showed significantly higher p21 protein levels than bleomycin-treated HS cultures (Figure 4G). We observed a negative correlation between the basal percentage of SA- β -GAL-positive cells and p53 activation after bleomycin treatment (R= −0. 6152, *P* < .0192), as well as between the percentage of basal SA-β-GAL-positive cells and p21 levels after bleomycin treatment (R= −0. 5646, *P* < .0345), suggesting that cultures with higher levels of senescence display an attenuated stress response (data not shown).

These findings suggest that the classification of primary VS cultures based on spontaneous SA-β-GAL activity correlates with differential activation of the p53/p21 pathway as revealed by bleomycin treatment. Despite similar levels of DNA damage induced by bleomycin, HS cultures exhibit attenuated p53/p21 signaling, which may underlie their lack of bleomycin-induced senescence.

### LS Primary Cultures Are Sensitive to Senolytic Targeting Following Bleomycin Treatment

**Figure 5.**
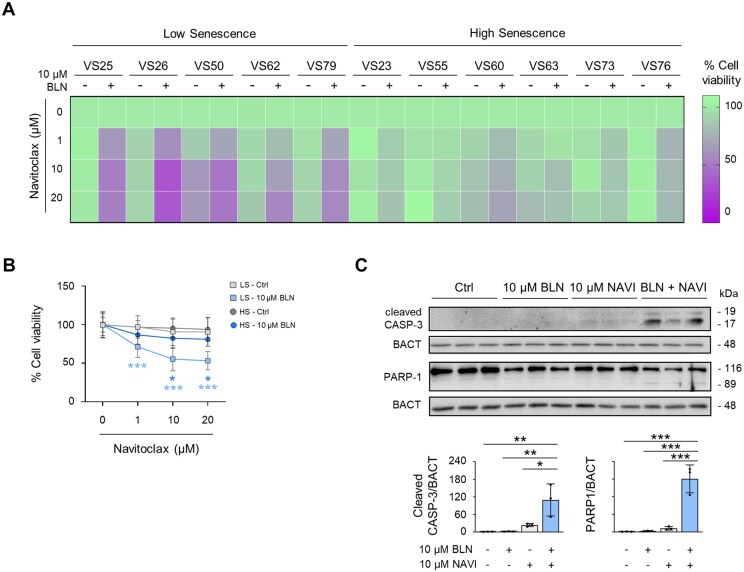
A one-two punch strategy induces apoptosis in LS-primary VS cultures. (A) Cell viability of control or bleomycin-treated (10 µM, 5 days) primary VS cells was assessed by crystal violet staining after treatment with 1, 10 or 20 µM navitoclax (24h). Percentage of primary VS viable cells are represented in a heatmap for the combined used concentrations, green-highest, magenta-lowest as illustrated at the right side of the figure. Data correspond to the mean of 3 independent primary cultures. (B) Data shown in A are plotted by clustering the different primary cell lines according to their senescence features: low senescence (LS: VS25, VS26, VS50, VS62, and VS79) and high senescence (HS: VS23, VS55, VS60, VS63, VS73, and VS76). Data correspond to the mean ± SD of 5 (LS) or 6 (HS) different cultures performed by triplicate. (C) Protein levels of active caspase 3 and proteolyzed PARP-1 were measured by western blotting in control and bleomycin-treated (10 µM, 5 days) primary cells (VS62) after navitoclax treatment (10 µM, 16h). β-actin was used as a loading control. Quantification of PARP-1 and CASP-3 protein levels found in VS62 primary cells is shown below. Values are normalized to β-actin. Data correspond to the mean ± SD of 3 independent cultures. Statistical significance was determined by two-way ANOVA. Symbols denote statistically significant differences between bleomycin treatment and control conditions: *P < .05; **P < .01; ***P < .001.

We next investigated whether the different subtypes of primary VS cultures could be selectively targeted using a combination of pro-senescence and senolytic agents. To this end, primary VS cultures were pretreated with bleomycin for 5 days, followed by a 24-hour exposure to increasing concentrations of the senolytic drug navitoclax (ABT-263). Cell viability was assessed using crystal violet staining. Navitoclax significantly reduced cell viability in primary VS cultures that had been pretreated with bleomycin, whereas navitoclax alone had no effect on any of the cultures analyzed (Figure 5A). Importantly, the reduction in viability following the combined treatment was more pronounced in LS-primary cultures (Figure 5B).

To further characterize this response, we performed western blotting analysis to assess apoptosis markers, including cleaved PARP-1 and activated caspase-3. These markers were upregulated in LS cultures following the combined treatment, confirming the induction of apoptosis observed in the viability assays (Figure 5C).

## Discussion

Spontaneous cellular senescence in cancer, independent of therapeutic intervention, remains a poorly understood phenomenon. Its presence has been documented in various tumor types, including ovarian and breast cancers, Hodgkin’s lymphoma, and murine models of ductal carcinoma in situ.^38^ In this study, we demonstrate that VS from patients who had not received prior radio- or chemotherapy contains a subset of SA-β-GAL-positive cells, indicative of a senescent phenotype. This observation is supported by transcriptomic profiling, which revealed a senescence-associated gene expression signature in VS tumors. Recent omics studies have mapped the cellular composition of VS tumors, revealing distinct VS cell subtypes.^39-43^ Further investigation will be required to define the transcriptional identity of senescent cells and to determine whether they correspond to any of the previously identified schwannoma cell subtypes.

The transcriptomic analysis of VS samples converges on a pattern consistent with key features of cellular senescence. At the level of pathway enrichment, cytokine signaling and inflammatory response pathways showed upregulation, while key regulators of cell cycle were markedly downregulated. At the gene level, a subset of genes were identified encompassing central regulators of cell cycle progression, including *FOXM1*, *CDK1*, *CDK2*, *E2F1, CHEK1, CCNB1,* and *CCNB2,* which were coordinately downregulated, together with genes involved in signaling, stress responses, and immune modulation (eg *TGFBR1*, *PI3KR1*, *FOXO1*, *NFATC2*, *IGFBP3,* and HLA class I genes), which were upregulated.^44-48^ Importantly, comparison with an independent dataset (Torres‑Martín et al.) revealed a consistent overlap in key upregulated and downregulated genes, including core cell cycle regulators and signaling components, supporting the reproducibility of this transcriptional pattern.^33^ These findings suggest that senescence is not merely a passive feature but may actively shape the biology of VS.

Our study also provides mechanistic insights into spontaneous senescence in primary VS cultures. Similar to other tumor types,^49^ VS cells exhibited canonical senescence markers, including SA-β-GAL activity, elevated p16 protein levels, and SASP gene expression. Importantly, the senescence phenotype varied among cultures, revealing a previously uncharacterized heterogeneity. Cultures with high spontaneous senescence (HS) showed reduced activation of the p53/p21 axis in response to DNA damage, despite comparable levels of γH2AX induction. This suggests that p16-driven senescence predominates in VS, as observed in normal SC during nerve regeneration,^50^ and rules out replicative senescence or culture-induced artifacts.^51^ The role of p16 as a tumor suppressor in VS is further supported by recent genetic studies linking *CDKN2A* mutations to sporadic VS and malignant transformation in vestibulocochlear nerve tumors.^52^^,^^53^ In addition to p16, HS cultures exhibited elevated expression of SASP components such as *IL1B, IL8, CCL2,* and *MMP3*, consistent with a pro-inflammatory microenvironment.^54^ While SASP factors can reinforce senescence, they also contribute to tumor progression and therapy resistance.^55^^,^^56^

Our findings underscore the functional consequences of senescence heterogeneity in VS. Cultures with low baseline senescence (LS) were more responsive to bleomycin-induced senescence and to senolytic treatment with navitoclax, which triggered apoptosis via the intrinsic pathway. In contrast, HS cultures were resistant to both interventions, likely due to a diminished p53/p21 signaling and altered apoptotic machinery.^57^^,^^58^ Activation of p53/p21 axis is essential for the senescence response to DNA damage induced by bleomycin. Our data demonstrated that senescence in VS cells is associated with a reduced activation of the p53/p21 axis, since neither p53 phosphorylation nor p21 protein levels were increased after DNA damage at the same extent in HS- than in LS-primary cultures, despite both groups increased in a similar extent γH2AX protein levels in response to bleomycin and presented similar basal p53 and p21 protein levels. In accord with these results, HS-primary cultures are also not able to significantly further increase the percentage of SA-β-GAL positive cells. Our findings agreed with recent reports that show that senescent cells manage to survive by limiting p53 activity through multiple mechanisms.^57^ In this regard, it has also suggested that VS show age-dependent deregulation of the p53 tumor suppressor gene.^58^ Our data suggest that inability of VS senescent cells to respond to genotoxic stress is compromised most likely due to inefficient p53 axis activation as occurs in senescent fibroblasts. It is also tempting to think that loss of p53 activity may enhance the expression of SASP components, contributing to therapy-induced senescence (TIS) resistance, as occurs in epithelial cells where it has been reported that functional loss of tumor suppressor p53 amplified SASP induced by TIS, which also contributes to chemotherapy resistance.^59^^,^^60^

Although navitoclax selectively eliminated bleomycin-treated LS cultures, it was ineffective against non-treated HS cultures, suggesting that spontaneous senescence may confer resistance to senolytic agents. This resistance could be due to mutations in BCL-2 family proteins, mitochondrial alterations, or metabolic rewiring.^61^ Alternative senolytics, such as flavonoids (fisetin, quercetin) or dasatinib-based combinations, may offer broader efficacy.^62^^,^^63^

Taken together, our data reveals that spontaneous cellular senescence is a biologically relevant and heterogeneous feature of VS. This heterogeneity influences the cellular response to DNA damage and senolytic therapies, providing a rationale for stratifying VS tumors based on senescence markers. The “one-two punch” strategy—combining senogenic and senolytic agents—may be effective in a subset of VS tumors with low spontaneous senescence, as demonstrated in HEI-193 cells.^28^ Although no correlation was found between senescence levels and clinical parameters (Supplementary Table S3), likely due to sample size limitations, this study offers a compelling proof of concept. Future research with larger cohorts is needed to validate these findings and explore their translational potential, including the design of biology-driven therapeutic strategies for VS.

In conclusion, this study advances our understanding of the biological heterogeneity of VS by identifying spontaneous cellular senescence as a defining and variable feature among tumors. Through integrated transcriptomic and functional analyses, we demonstrate that senescence in VS is primarily driven by p16 upregulation and SASP amplification, rather than by canonical p53/p21-mediated pathways. Importantly, the degree of spontaneous senescence correlates with differential sensitivity to DNA damage and senolytic agents, revealing distinct cellular subtypes with implications for therapeutic stratification. These findings underscore the relevance of senescence profiling in VS and support the development of biology-driven approaches to improve experimental modeling and guide future pharmacological interventions.

## Supplementary Material

vdag190_Supplementary_Data

## Data Availability

Datasets generated during the study can be found at the following addresses: https://doi.org/10.20350/digitalCSIC/17249; https://doi.org/10.20350/digitalCSIC/17538
