# Supplementary material for: Spontaneous cellular senescence and stress response heterogeneity in human vestibular schwannomas
Source: Neurooncol Adv. 2026 Jul 22;8(1):vdag190. doi: 10.1093/noajnl/vdag190 (PMC13451090; doi:10.1093/noajnl/vdag190)
Supplement: Supplementary file 1 [file vdag190_supplementary_data.zip › Supplementary Table-1_NOA-D-25-00427R1R1.docx]

**Supplementary table 1. Antibodies used for western blotting and/or immunofluorescence.**

| **Target** | **Dilution** | **Technique** | **Manufacturer** | **Catalog Nº** |
| --- | --- | --- | --- | --- |
| β-actin | 1:100000 | WB | Sigma | #A3854 |
| Cleaved Caspase-3 (175 Asp) | 1:1000 | WB | Cell Signaling Technology | #9661 |
| PARP-1 (h-250) | 1:1000 | WB | Santa Cruz | #sc-7150 |
| Phospho-Histone H2A.X (Ser139) | 1:1000 | WB | Cell Signaling Technology | #2577 |
|  | 1:400 | IF |  |  |
| Phospho-p53 (Ser15) | 1:1000 | WB | Cell Signaling Technology | #9284 |
| p16 | 1:1000 | WB | Abcam | #ab108349 |
| p21 Waf1/Cip1 (12D1) | 1:1000 | WB | Cell Signaling Technology | #2947 |
| p53 | 1:500 | WB | Santa Cruz | #sc-99 |
| Vinculin | 1:20000 | WB | Santa Cruz | #sc-73614 |
| HRP-anti-mouse IgG | 1:3000 | WB | Bio-Rad laboratories | #1706516 |
| HRP-anti-rabbit IgG | 1:3000 | WB | Bio-Rad laboratories | #1706515 |
| Goat-anti-rabbit IgG (AF546) | 1:300 | IF | Invitrogen | #A11035 |

Technical information of antibodies used includes dilution, application, brand and product number. WB: Western blotting; IF: immunofluorescence.
