# Supplementary material for: Spontaneous cellular senescence and stress response heterogeneity in human vestibular schwannomas
Source: Neurooncol Adv. 2026 Jul 22;8(1):vdag190. doi: 10.1093/noajnl/vdag190 (PMC13451090; doi:10.1093/noajnl/vdag190)
Supplement: Supplementary file 1 [file vdag190_supplementary_data.zip › Supplementary Table-2_NOA-D-25-00427R1R1.docx]

**Supplementary Table 2. KEGG pathway enrichment analysis results.**

| **GENE NAME** | **FC** | **q-value** | **FUNCTION** | **CELLULAR SENESCENCE** |
| --- | --- | --- | --- | --- |
| **Control of cell cycle** |  |  |  |  |
| *CCNB1*  (cyclin B1) | -5.841 | 0.002 | G_2_/M transition | Inhibitor^1^ |
| *CDK1*  (cyclin dependent kinase 1) | -5.59 | 0.004 | Modulates centrosome cycle and mitotic onset, promotes G_2_/M transition, regulates G_1_ progress and G_1_/S transition | Inhibitor^2^ |
| *CDK2*  (cyclin dependent kinase 2) | -3.12 | 0.010 | Essential for meiosis. G_1_/S transition | Inhibitor. Related to oncogene, replicative and stress-induced senescence^3^ |
| *E2F1*  (E2F transcription factor 1) | -4.04 | 0.010 | Control of cell cycle and action of tumor suppressor proteins. It can mediate cell proliferation and apoptosis | Inhibitor^4^ |
| *CCND2*  (cyclin D2) | 2.98 | 0.025 | G_1_/S transition | Inhibitor. Related to oxidative stress-induced senescence^5^ |
| *FOXM1*  (FOXM1 transcription factor) | -5.21 | 0.028 | Essential for mitosis | Inhibitor^6^ |
| *CHEK1*  (checkpoint kinase 1) | -4.23 | 0.033 | Required for checkpoint mediated cell cycle arrest in response to DNA damage or the presence of unreplicated DNA. Important for DNA damage responses | Inhibitor or inducer depending on the activation period. Stress-induced senescence^7^ |
| *CCNB2*  (cyclin B2) | -5.70 | 0.040 | G_2_/M transition  SASP modulator | Dual function: inhibitor^8^ and inducer^9^ |
| **Cellular damage and stress** |  |  |  |  |
| *SMAD3*  (SMAD family member 3) | -2.92 | 0.002 | TGF-beta pathway. Transmits signals from surface to the nucleus regulating gene activity and proliferation. Tumor suppressor. Inhibitory effect on wound healing | Inductor^10^ |
| *MAP2K3*  (mitogen-activated protein kinase 3) | -2.36 | 0.007 | Phosphorylates MAPK14/p38-MAPK | Inhibitor^2^ or inducer^11^ depending on the context |
| *TP53*  (tumor protein p53) | -2.701 | 0.018 | Tumor supressor. Inductor of growth arrest or apoptosis | Inducer. Related to oncogene, replicative and stress-induced senescence^12^ |
| *MAPK12* | -4.33 | 0,021 | Triggers a cascade of cellular responses caused by pro-inflammatory cytokines or physical stress | Inducer^13^ |
| *TGFBR1*  (transforming growth factor beta receptor 1) | 1.63 | 0.035 | Transduction of TGF-beta signal to the cytoplasm | Inducer^14^ |
| *NRAS*  (NRAS proto-oncogen, GTPase) | -1.42 | 0.045 | GTPase activity | Inducer. Related to oncogene-induced senescence^15^ |
| **Calcium signaling** |  |  |  |  |
| *ITPR1*  (inositol 1,4,5-triphosphate receptor type 1) | 4.50 | 0.002 | Mediates calcium release from the ER. Plays a role in ER-stress induced apoptosis | Inducer. Related to oncogene-induced senescence^16^ |
| *CAPN2* (calpain 2) | -2.05 | 0.002 | Catalyzes limited proteolysis of substrates involved in cytoskeletal remodeling and signal transduction / Overexpressed in ageing humans | ND |
| *CACNA1D*  (calcium voltage-gated channel subunit alpha 1D) | 3.73 | 0.005 | Mediates calcium influx in response to depolarization. Regulates intracellular processes such as contraction, secretion, neurotransmission and gene expression | Inducer via the inhibition of autophagy^17^ |
| *NFATC2*  (nuclear factor of activated T cells 2) | 3.41 | 0.007 | Transcription factor integral for the development and function of the immune system. Plays a role in the inducible expression of cytokine genes in T-cells, especially in the induction of the IL-2, IL-3, IL-4, TNF-alpha or GM-CSF. Promotes invasive migration through the activation of GPC6 expression and WNT5A signalling | SASP modulator^18^ |
| **Immunity** |  |  |  |  |
| *HLA-E*  (major histocompatibility complex, class I, E) | 2.465 | 0.006 | Innate and adaptative immune responses. Modulates natural killer and cytotoxic T lymphocyte activation and inhibitory function | Protective effect against the elimination of senescent cells^19^ |
| *HLA-F*  (major histocompatibility complex, class I, F) | 4.37 | 0.014 | Immune surveillance, tolerance and inflammation | ND |
| **Metabolic survival** |  |  |  |  |
| *PI3KR1*  (phosphoinositide-3-kinase regulatory subunit 1) | 3.63 | 0.002 | Necessary for insulin-stimulated increase in glucose uptake and glycogen synthesis in insulin-sensitive tissues. Plays an important role in signalling in response to FGFR1, FGFR2, FGFR3, FGFR4, KITLG/SCF, KIT, PDGFRA and PDGFRB. Role in ITGB2 signalling. Cellular response to ER stress | Inhibitor. Oxidative stress-induced senescence^20^ |
| *FOXO1*  (forkhead box O1) | 3.82 | 0.002 | Not determined. May play a role in myogenic growth and differentiation | Inducer^21^ |
| *HIPK3*  (homeodomain interacting protein kinase 3) | 2.47 | 0.002 | Transcription regulation, apoptosis, steroidogenic gene expression. Seems to negatively regulate apoptosis by promoting FADD phosphorylation | ND |
| *EIF4EBP1* (eukaryotic translation initiation factor 4E binding protein 1) | -3.56 | 0.002 | Mediates the regulation of protein translation by hormones, growth factors and other stimuli (MAPK / Mtorc1 pathways) | Inducer. Oncogene-induced senescence^22^ |
| *IGFBP3*  (insulin like growth factor binding protein 3) | 2.64 | 0.016 | IGF-binding proteins prolong the half-life of the IGFs and have been shown to either inhibit or stimulate the growth promoting effects of the IGFs on cell culture. They alter the interaction of IGFs with their cell surface receptors. Also exhibits IGF-independent antiproliferative and apoptotic effects mediated by its receptor | Inducer. Related to stress-induced senescence^23^ |
| *SLC25A6*  (solute carrier family 25 member 6) | -1.68 | 0.027 | Implicated in the function of the permability transition pore complex (PTPC), which regulates the release of mitochondrial products that induce apoptosis | ND |
| *SLC25A5*  (solute carrier family 25 member 5; ANT2) | -1.47 | 0.047 | Supressed expression induces apoptosis and inhibits tumor growth | Inhibitor. Related to stress-induced senescence^14^ |

Twenty-seven differentially expressed genes (DEGs) were identified between eleven vestibular schwannoma (VS) (VS2, VS4, VS17, VS20, VS21, VS22, VS24, VS25, VS27, VS30 and VS31) and Schwann cells (SC). Pathway enrichment analysis from these genes was performed, specifically filtering for the KEGG Senescence pathway. The genes are grouped into pathways and ordered according to increasing q-values. The function and whether they are inducers or inhibitors of cellular senescence are also described. KEGG = Kyoto encyclopedia of genes and genomes. ND = not described.

**References**

1. Zhang H, Zhang X, Li X, et al. Effect of CCNB1 silencing on cell cycle, senescence, and apoptosis through the p53 signaling pathway in pancreatic cancer. *J Cell Physiol*. 2018;234(1). doi:10.1002/jcp.26816
2. Lahtela J, Corson LB, Hemmes A, et al. A high-content cellular senescence screen identifies candidate tumor suppressors, including EPHA3. *Cell Cycle*. 2013;12(4). doi:10.4161/cc.23515
3. Zalzali H, Nasr B, Harajly M, et al. CDK2 transcriptional repression is an essential effector in p53-dependent cellular senescence - Implications for therapeutic intervention. *Molecular Cancer Research*. 2015;13(1). doi:10.1158/1541-7786.MCR-14-0163
4. Xie Q, Peng S, Tao L, Ruan H, Yang Y, Li T. M, Adams U, Meng S, Bi X, Dong M. Q, & Yuan Z. *The* E2F transcription factor 1 regulates cellular and organismal senescence by inhibiting Forkhead box O transcription factors. *Journal of biological chemistry.* 2014; *289*(49), 34205–34213. doi:10.1074/jbc.M114.587170
5. Xu, X., Kim, J. J., Li, Y., Xie, J., Shao, C., & Wei, J. J. (2018). Oxidative stress-induced miRNAs modulate AKT signaling and promote cellular senescence in uterine leiomyoma. Journal of molecular medicine (Berlin, Germany), 96(10), 1095–1106. https://doi.org/10.1007/s00109-018-1682-1
6. Park HJ, Carr JR, Wang Z, Nogueira V, Hay N, Tyner AL, Lau LF, Costa RH, Raychaudhuri P. FoxM1, a critical regulator of oxidative stress during oncogenesis. EMBO J. 2009 Oct 7;28(19):2908-18. doi: 10.1038/emboj.2009.239. Epub 2009 Aug 20. PMID: 19696738; PMCID: PMC2760115.
7. Poehlmann A, Habold C, Walluscheck D, et al. Cutting edge: Chk1 directs senescence and mitotic catastrophe in recovery from G2 checkpoint arrest. *J Cell Mol Med*. 2011;15(7). doi:10.1111/j.1582-4934.2010.01143.x
8. Wu S, Su R, Jia H. Cyclin B2 (CCNB2) Stimulates the Proliferation of Triple-Negative Breast Cancer (TNBC) Cells *In Vitro* and *In Vivo*. Dis Markers. 2021 Jul 26;2021:5511041. doi: 10.1155/2021/5511041. PMID: 34354775; PMCID: PMC8331305.
9. Wang Y, Zhang H, Wang M, et al. Ccnb2/sasp/cathepsin b & pge2 axis induce cell senescence mediated malignant transformation. *Int J Biol Sci*. 2021;17(13). doi:10.7150/ijbs.63430
10. Bryson BL, Junk DJ, Cipriano R, Jackson MW. STAT3-mediated SMAD3 activation underlies Oncostatin M-induced Senescence. *Cell Cycle*. 2017;16(4). doi:10.1080/15384101.2016.1259037
11. Jia M, Souchelnytskyi N, Hellman U, O'Hare M, Jat PS, Souchelnytskyi S. Proteome profiling of immortalization-to-senescence transition of human breast epithelial cells identified MAP2K3 as a senescence-promoting protein which is downregulated in human breast cancer. Proteomics Clin Appl. 2010 Nov;4(10-11):816-28. doi: 10.1002/prca.201000006. PMID: 21137025.
12. Lin AW, Barradas M, Stone JC, van Aelst L, Serrano M, Lowe SW. Premature senescence involving p53 and p16 is activated in response to constitutive MEK/MAPK mitogenic signaling. Genes Dev. 1998 Oct 1;12(19):3008-19. doi: 10.1101/gad.12.19.3008. PMID: 9765203; PMCID: PMC317198.
13. Ferrand, M., Kirsh, O., Griveau, A., Vindrieux, D., Martin, N., Defossez, P. A., & Bernard, D. (2015). Screening of a kinase library reveals novel pro-senescence kinases and their common NF-κB-dependent transcriptional program. Aging, 7(11), 986–1003. https://doi.org/10.18632/aging.100845
14. Kretova M, Sabova L, Hodny Z, et al. TGF-β/NF1/Smad4-mediated suppression of ANT2 contributes to oxidative stress in cellular senescence. *Cell Signal*. 2014;26(12). doi:10.1016/j.cellsig.2014.08.029
15. Zhu H, Chan ASL, Narita M. The rise of RAS: how gradual oncogene activation shapes the OIS spectrum. Genes Dev. 2025 Aug 1;39(15-16):936-947. doi: 10.1101/gad.352761.125
16. Wiel C, Lallet-Daher H, Gitenay D, et al. Endoplasmic reticulum calcium release through ITPR2 channels leads to mitochondrial calcium accumulation and senescence. *Nat Commun*. 2014;5. doi:10.1038/ncomms4792
17. Fan P, Pu D, Lv X, Hu N, Feng X, Hao Z, Sun Y, He L. Cav 1.3 damages the osteogenic differentiation in osteoporotic rats by negatively regulating Spred 2-mediated autophagy-induced cell senescence. J Cell Mol Med. 2020 Dec;24(23):13863-13875. doi: 10.1111/jcmm.15978. Epub 2020 Oct 30. PMID: 33124763; PMCID: PMC7754063.
18. Chandrasekaran A, Lee MY, Zhang X, Hasan S, Desta H, Tenenbaum SA, Melendez JA. Redox and mTOR-dependent regulation of plasma lamellar calcium influx controls the senescence-associated secretory phenotype. Exp Biol Med (Maywood). 2020 Nov;245(17):1560-1570. doi: 10.1177/1535370220943122. Epub 2020 Jul 19. PMID: 32686475; PMCID: PMC7787549.
19. Pereira BI, Devine OP, Vukmanovic-Stejic M, et al. Senescent cells evade immune clearance via HLA-E-mediated NK and CD8+ T cell inhibition. *Nat Commun*. 2019;10(1). doi:10.1038/s41467-019-10335-5
20. Zhang P, Zhang N, Hu Y, et al. Role of PI3K/AKT/MAOA in glucocorticoid-induced oxidative stress and associated premature senescence of the trabecular meshwork. *Aging Cell*. 2024;00. doi:10.1111/acel.14452
21. Latorre E, Ostler EL, Faragher RGA, Harries LW. FOXO1 and ETV6 genes may represent novel regulators of splicing factor expression in cellular senescence. *FASEB Journal*. 2019;33(1). doi:10.1096/fj.201801154R
22. Petroulakis E, Parsyan A, Dowling RJO, et al. p53-Dependent Translational Control of Senescence and Transformation via 4E-BPs. *Cancer Cell*. 2009;16(5). doi:10.1016/j.ccr.2009.09.025
23. Debacq-Chainiaux F, Pascal T, Boilan E, Bastin C, Bauwens E, Toussaint O. Screening of senescence-associated genes with specific DNA array reveals the role of IGFBP-3 in premature senescence of human diploid fibroblasts. *Free Radic Biol Med*. 2008;44(10). doi:10.1016/j.freeradbiomed.2008.02.001
