# Supplementary material for: Spontaneous cellular senescence and stress response heterogeneity in human vestibular schwannomas
Source: Neurooncol Adv. 2026 Jul 22;8(1):vdag190. doi: 10.1093/noajnl/vdag190 (PMC13451090; doi:10.1093/noajnl/vdag190)
Supplement: Supplementary file 1 [file vdag190_supplementary_data.zip › Supplementary Table-3_NOA-D-25-00427R1R1.docx]

**Supplementary Table 3. Patient demographics and clinical parameters for low and high senescence primary cell cultures derived from vestibular schwannomas.**

| Characteristic | Low Senescence | High Senescence |
| --- | --- | --- |
| Age, year |  |  |
| Mean ± SD | 48.29 ± 15.01 | 51.57 ± 16.93 |
| Sex |  |  |
| Male | 3 | 2 |
| Female | 4 | 5 |
| Recurrence of previous tumor | 0 | 0 |
| Koos gradient scale |  |  |
| 1 | 0 | 0 |
| 2 | 1 | 0 |
| 3 | 0 | 1 |
| 4 | 6 | 6 |
| Preoperative audition |  |  |
| Serviceable hearing (A/B) | 1 | 4 |
| Non-serviceable hearing (C/D) | 6 | 3 |
| Tumoral size (diameter, mm) | 28.93 ± 10.73 | 30.33 ± 6.86 |
| Tumoral size (cc) | 9.69 ± 7.81 | 12.81 ± 6.74 |
| Cystic appearance on MRI | 2 | 2 |
| Previous radiotherapy | 0 | 0 |
| NF2-SWN | 1 | 0 |
| *NF2* status (blood) |  |  |
| N/A | 5 | 0 |
| ND | 0 | 2 |
| Negative | 2 | 5 |
| *NF2* status (tissue) |  |  |
| N/A | 1 | 0 |
| ND | 2 | 3 |
| Negative | 1 | 0 |
| Pathogenic variant | 4 | 4 |
| Likely pathogenic variant | 1 | 1 |

Clinical information of patients diagnosed with vestibular schwannoma (VS), categorized by the level of senescence of the primary cultures from which they were derived: low senescence (LS: VS25, VS26, VS50, VS62, VS78, VS79 and VS80) and high senescence (HS: VS23, VS55, VS60, VS63, VS72, VS73 and VS76). The table includes age at diagnosis, gender, Koos gradient scale, preoperative hearing, tumor measurements and volume, cystic appearance, previous treatment with radiotherapy, diagnose with *NF2* related Schwannomatosis, and *NF2* status in blood and tissue. Koos gradient scales classifies VS based on extramental extension and compression of the brainstem: 1, small intracanicular tumor; 2, small tumor with protrusion into de cerebellopontine cistern, no contact with the brainstem; 3, tumor occupying the cerebellopontine cistern with no brainstem displacement; 4, large tumor with brainstem and cranial nerve displacement. Preoperative hearing was classified according to the American Academy of Otolaryngology–Head and Neck Surgery into four grades based on pure tone average (PTA) and the speech discrimination score (SDS): A, PTA < 30 dB, SDS 70-100%; B, PTA < 31-50 dB, SDS 50-69%; C, PTA > 50 dB, SDS 50-69%; D, any PTA, SDS < 50%. Tumor volume was calculated on Magnetic Resonance Imaging (MRI). Variant classification was performed according to ACMG recommendations. ND: not determined; N/A: not available.
